# Editorial: Impact of virtual reality on sensory neuroscience: delving into body ownership and agency

**DOI:** 10.3389/fnhum.2025.1668123

**Published:** 2025-08-04

**Authors:** Maria Pyasik, Maurice Lamb, Adriana Salatino

**Affiliations:** ^1^Laboratory of Cognitive Neuroscience, Department of Languages and Literatures, Communication, Education and Society, University of Udine, Udine, Italy; ^2^Department of Informatics, University of Skövde, Skövde, Sweden; ^3^Department of Life Sciences, Royal Military Academy, Brussels, Belgium

**Keywords:** body representation, virtual reality, body ownership, embodied cognition, cognitive neuroscience

## Introduction

In the past decade, immersive virtual reality (IVR) tools have rapidly developed in cognitive neuroscience (Slater, 2018), particularly relating to body representation studies. The widespread adoption of these tools results from technical advances in IVR, along with the ability to create a strong experience of owning (embodying) a virtual body and of controlling it as one's own in virtual environments (Slater et al., 2009). The flexibility of virtual embodiment scenarios allows for body representation research to expand beyond the mechanisms of embodiment and to explore more deeply the higher-order effects of embodiment on cognition and action. Indeed, there is a growing body of literature demonstrating that embodiment of virtual avatars, their movements and/or features affect multiple domains of cognition, impacting perception, affective processes, and motor functioning (Pyasik et al., 2022).

While the existing evidence offers various possibilities of practical applications of virtual embodiment, including in clinical and rehabilitation settings (Portingale et al., 2024; Tambone et al., 2021a), it is still not clear what the mechanisms and the constraints of these higher-order effects are. Indeed, they are rarely investigated systematically and beyond their behavioral indices (Pyasik et al., 2022).

## Research Topic content

The present Research Topic aims to highlight the potential of IVR, focusing on virtual embodiment, for investigating a range of motor and cognitive processes. The four articles included in this collection cover different domains, namely the interplay of the sense of agency and body ownership in IVR, sensorimotor learning in rehabilitation contexts, body-related implicit attitudes, and the role of virtual human agents in spatial navigation and learning.

Virtual embodiment includes two key aspects: body ownership, i.e., the feeling that a virtual avatar is one's own body, and the sense of agency over its movements (Slater et al., 2009). While both are crucial for embodiment, there is a double dissociation between them in terms of their constraints and mechanisms (Kalckert and Ehrsson, 2014, 2012; Pyasik et al., 2018). To address this issue in the novel context of the IVR techniques, the systematic review by Girondini et al. provides an analysis of the conditions and constraints of IVR paradigms that targeted the implicit measures of body ownership and sense of agency. Their results support the idea of the double dissociation and an interplay of the two components of embodiment even in the virtual environments, and allow grouping the IVR manipulations into those selectively affecting body ownership (e.g., manipulating the appearance of the avatar) or the sense of agency (e.g., manipulating the extent of movement control). Capitalizing on the possibility to synchronize participants' movements with certain changes in the virtual environment, and on the fact that such synchrony enhances the sense of agency, as highlighted in Girondini et al., the study by Huang et al. investigated the effects of visual-only assistance in a VR-based motor training. Comparing two types of continuous visual assistance—the direct shift of an object toward the target and the less direct correction of the trajectory controlled by the participant—showed that neither of the types was beneficial for the short-term motor learning. Interestingly, when the visual-only cues concern the movements of an embodied avatar in IVR, the motor system is activated and the motor functioning might be improved (Álvarez de la Campa Crespo et al., 2023), which highlights the central role of the presence of a virtual body in VR-based motor learning and rehabilitation paradigms. Beyond the embodiment of movements, another crucial aspect of virtual embodiment studies concerns embodiment of avatars' features that subsequently affects participants' implicit attitudes (Pyasik et al., 2022). Crucially, embodiment occurs independently of the discrepancies between the avatar's and the participant's appearance (Pyasik et al., 2022). However, while multiple studies investigated the effects of embodying differently-looking avatars, they typically did not customize them for each participant. The study by Gonzalez-Franco et al. aimed at filling this gap by investigating the effects of customizing the avatar's body size on the subsequently measured implicit weight bias. Their findings confirmed that, in the first-person perspective, embodiment is equally strong for the avatars of one's own body size and of a larger one, and that the latter results in a recalibration of the perceived own body size (Tambone et al., 2021b; Preston and Ehrsson, 2014). However, implicit weight bias was not modulated by embodiment of a larger avatar, in line with (Tambone et al., 2021b). Overall, this study further supports the idea that embodiment is largely appearance-independent (Tsakiris, 2010; Pyasik et al., 2020). Nonetheless, avatar customization might be investigated further in the context of user satisfaction and levels of virtual scenarios' engagement. Finally, virtual scenarios might include multiple virtual characters (avatars) in addition to that of the participant. This creates the possibility to study social interactions in controlled environments (e.g., Banakou et al., 2020; Hasler et al., 2017), as well as the role of the presence of (virtual) human agents in various contexts. The study by Sánchez Pacheco et al. investigated the role of virtual agents in spatial navigation and learning in a virtual city. Their results demonstrated that the presence of virtual agents affected the navigation patterns during exploration. The subsequent spatial recall of the elements of the environment (buildings) benefitted from the presence of virtual agents, while the opposite pattern (the presence of building affecting the recall of the agents) was not shown. Importantly, the beneficial effect of the agents' presence was further enhanced when the agents were contextually incongruent. These findings highlight the importance the presence, and the contextual congruency, of agents in virtual settings that concern spatial navigation/learning, as well as in broader contexts of multi-character virtual environments.

## Outlook

The articles in this Research Topic highlighted the use of the IVR across multiple domains, with the focus on the effects of embodying virtual avatars. The wide variety of investigated processes further confirms the usefulness of the IVR in cognitive neuroscience research. They also underline the need to develop standardized experimental protocols and measurements to enhance the replicability and generalizability of findings within both research and applied contexts.
